# Clinical features of extra-uterine pregnancy in Cameroon: a review of 148 cases at the Yaounde Central Hospital

**Published:** 2018-09

**Authors:** JH Fouedjio, YF Fouelifack, TJ Fouogue, LM Kana, ER Mbu

**Affiliations:** Department of Obstetrics and Gynecology of the Yaounde Central Hospital. PO Box 83 Yaounde - Cameroon; Faculty of Medicine and Biomedical Sciences of the University of Yaounde 1. PO box 1364 Yaounde – Cameroon; Yaounde Higher Institute of Medical Technologies. PO Box 31186 Yaounde - Cameroon; Department of Obstetrics and Gynecology of the Bafoussam Regional Hospital, PO Box 980 Bafoussam, Cameroon.

**Keywords:** Extra-Uterine Pregnancy, amenorrhea, salpingectomy, methotrexate, Ectopic pregnancy

## Abstract

**Background:**

Extra-uterine pregnancy (EUP) is frequent in Cameroon but little is known about its clinical features.

**Objective:**

To describe the epidemiological, diagnostic, therapeutic and prognostic features of extra-uterine EUPs at the Yaounde Central Hospital in Cameroon.

**Methods:**

We carried out a cross-sectional study from the 1 st of January 2015 till January 31 st 2016 at the maternity of the Yaounde Central Hospital. All patients admitted for EUP were included. Data was analysed with Epi info 3.5.4 and SPSS 2.0.

**Results:**

In this study, EUPs were diagnosed between 7 and 11 weeks of amenorrhoea including 148 cases (3.14%) out of 4707 deliveries.The mean age of patients was 28.54 ± 5.85 years and participants were mainly single (80.4%), self-employed (48.7%) and for most living in town (80.4%). Half of the patients (50.0%) had history of sexually transmitted infections and 56.8% have had a voluntary abortion. Male condom was the most frequently used contraceptive (54.7%) and EUP was iterative in 8.8% of cases. Most EUPs were ruptured (85%) and some presented haemorrhagic shock (67.5%) with nearly all (93.7%) requiring management by laprotomic salpingectomy. Death occurred in 1.3% (2/148) of cases.

**Conclusion:**

In Yaounde - Cameroon, diagnosis and medical care of EUP takes place with a cosiderable delay leading to high risk complications. Salpingectomy via laparotomy remains the mainstay for management of EUP.

## Introduction

Extra-uterine pregnancy (EUP) is the main cause of first trimester maternal mortality (Randriambololona et al., 2012). Besides being life-threatening, due to ruptures with massive hemoperioneum, late diagnosis is also a common factor in resource-challenged settings. Moreover, EUP is correlated to infertility. Indeed, one third of EUP cases occur in nulliparous women, half of whom will remain sterile (Balde et al., 2014). Features of EUP in Cameroon have not been extensively studied (Foumane et al., 2011; Kamga et al., 2017; Dohbit et al., 2010) thus, our goal is to determine the epidemiology, diagnostic, therapeutic and prognostic features of EUP in Yaounde.

## Methods and Materials

### Study design

We conducted a descriptive cross-sectional study at the maternity of the Yaounde Central Hospital. The study was carried out from January 1 st , 2015 till January 31 st , 2016. The population studied consisted of pregnant women consulting at maternity services at the beginning of pregnancy. Those diagnosed with ectopic pregnancies were included after informed consent. A minimum sample size of 63 participants was determined by the Lorentz’s formula.

### Data collection

Data was collected from all patients participating in this study and included sociodemographic (age, occupation, marital status) and obstetrical characteristics (gestational age and parity), medical history (pelvic infections, abortions, abdominal of pelvic surgery, assisted reproductive techniques, tobacco smoking, contraception, chronic pelvic pain, extra-uterine pregnancy, Human Immuno-Deficiency Virus status), clinical parameters (signs and symptoms, site of the EUP) and modality of treatment.

### Statistics

Data management was done with Epi info version 3.5.4 and SPSS 2.0. Quantitative data were presented as mean and qualitative data percentages.

### Ethics

Ethical clearance was obtained from the institutional ethical board of the faculty of Medicine of the University of Douala. We also obtained an administrative clearance from the executives of the Yaounde Central hospital. Prior to their inclusion, all participants signed an informed consent form.

## Results

### Socio-demographics and obstetrical features

A total of 148 EUPs were recorded over a total of 4707 deliveries (3.1%). The mean age for EUPs was 28.5 ± 5.9 years. Most patients (81.8%) were aged between 20 and 34 years old. There was a predominance of married women among (80.4%) and a vast majority of these patients living in urban areas. Approximately half (48.7%) of our patients were self-employed. More than half (52.7%) of these patients were either nullipara (27.7%) or primipara (25.0%) while grand multipara (Gravida 5 and above) accounted for 8.1% (12/148). Eighty (54%) patients didn’t know their HIV status. Forty one patients (27.7%) had no contraceptive method. Rupture occurred between 7 and 12 weeks in 68.4% of patients. History of induced abortion was the most frequent risk factor (56.8%) followed by pelvic inflammatory disease (50.0%). Only 8 patients (5.4%) had previously undergone pelvic surgery. Tobacco consumption (smoking) was found in 13 patients (8.8 %). The sociodemographic and obstetrical parameters of participants are summarized in Tables I and II.

**Table I:** Sociodemographic features of patients with EUP.

Variables	N (%)
Age	
≤ 15	1 (0.7)
16-19	7 (4.7)
20-24	27 (18.2)
25-29	57 (38.6)
30-34	37 (25.0)
35-39	12 (8.1)
≥40	7 (4.7)
Marital status	
Single	119 (80.4)
Married	26 (17.5)
Divorced	2 (1.4)
Widow	1 (0.7)
Occupation	
Housewife	28 (18.9)
Civil servant	20 (13.5)
Student	28 (18.9)
Self-employed	72 (48.7)
Area of residence	
Rural	29 (19.6)
Urban	119 (80.4)

**Table II:** Obstetrical features of women with EUP.

Variables	N (%)
Parity	
0	41 (27.7)
1	37 (25.0)
2	29 (19.6)
3 – 4	29 (19.6)
≥ 5	12 (8.1)
*HIV status	
Positive	11 (38.5)
Negative	57 (7.4)
Unknown	80 (54.1)
Contraception	
Condom	81 (54.7)
None	41 (27.7)
Hormonal	36 (24.3)
Intra Uterine Device	1 (0.7)
Gestational age in weeks (based on *LMP)	
Unknown (forgotten *LMP)	8 (5.4)
≤ 6	25 (16.8)
7 – 12	101(68.4)
≥ 12	14 (9.4)
Risk factors of **EUP	
Sexually transmitted infections	74 (50.0)
Tobacco smoking	13 (8.8)
Pelvic surgery	8 (5.4)
Chronic pelvic pain	20 (13.5)
History of EUP	13 (8.8)
Medical treatment	11 (84.6)
Surgical management	2 (15.4)
History of abortion	
Induced	84 (56.8)
Spontaneous	9 (6.1)
	10

### Clinical features

In patients presenting EUPs, abrupt ruptures happened in 128 (86.4%) of them. These patients had amenorrhea out of which 99 (67.5%) of them reported pelvic pain. One hundred (67.5%) patients presented with hemodynamic shock due to the EUP. In our series, EUPs were implanted in the fallopian tube in 142 (95.9%) patients and implantation in the ampulla was the most common site of implantation (115 cases). The clinical features presented in this section are summarized in table III.

**Table III:** Clinical features of women with EUPs.

Variables	N (%)
Clinical signs	
Amenorrhoea	148 (100.0)
Pelvic pain	99 (67.0)
Metrorrhagia	2 (1.0)
Signs of hemodynamic shock	100 (67.5)
Sites of *EUPs	
Ampullary	115 (77.7)
Isthmic	20 (13.5)
Intramural	6 (4.0)
Abdominal	6 (4.0)
Infudibular	1 (0.7)

*EUP : Extra Uterine Pregnancy. EUPs were ruptured in 86.4% of cases.

### Therapeutic aspects

The technique for management of UPSs was surgical laparotomy in 139 (93.9%) patients. In the practice, the following procedures were carried out: total salpingectomy in 133 (93.7%) cases; tubal conservation after milking in 4 (2.8%) cases and partial salpingectomy in 5 (3.5%) cases. Management was medical (intramuscular methotrexate) in 9 cases of which 6 were successful. Blood transfusion was carried out for 125 (84.5%) patients. Two deaths were registered (1.3%).

## Discussion

An overall EUP rate of 3.1% was found in Yaounde. This figure is similar to those reported in different locations in Cameroun a decade ago: 2.3% in Bafoussam (Dohbit et al., 2010), 3.4% in Sangmelima (Kenfack et al., 2012) and 4.2% in Yaounde (Foumane et al., 2011) but also to those reported in other African countries and in Europe (Randriambololona et al., 2012; Balde et al., 2014; Florin-Andrei et al. 2015, Perrine et al., 2014). In our series, the mean age of patients was 28.5 ± 5.9 years. This is similar to the overall mean age reported for different locations in Africa: 28.6 years in Conakry-Guinea, 29.6 years in Bafoussam-Cameroun, (Dohbit et al. 2010), 26.5 years in Sangmelima-Cameroun (Kenfack et al., 2012) and 30.7 years in Madacasgar (Randriambololona et al., 2012). These rates are lower than those reported by Rana et al. in India where variation from 1.4% at 21 years up to 6.9% at 44 years has been reported (Rana et al., 2013). This is in compliance with a setting such as Cameroon Cameroon because most urban women plan their first childbirth between 20 and 30 years old as demonstrated by the overwhelming predominance of a parity ≤ 2. Interestingly, we noted that EUP was mostly found in single women. This observation differs from several reports in other African countries (Randriambololona et al., 2012; Balde et al., 2014; Dembele et al., 2006; Ali et al., 2016; Elyounsi et al., 2009; Siham et al., 2016). Thus the question remains, does this implies that single women in our settings are more exposed to risk factors than their married counterparts? This issue has not yet been explored in Cameroon.

Furthermore, in our study, the mean gestational age at the time of EUP diagnosis was similar to those commonly reported in the literature (Randriambololona et al., 2012; Kenfack et al., 2012). The most frequent risk factor was a history of induced abortion, in-line with what has been reported in Madagascar (Randriambololona et al., 2012). History of sexually transmitted infections which is the most important risk factor of EUP ranked second. Balde et al., 2014 in Conakry-Guinea reported similar observations. Indeed, it has been proven that reducing the prevalence of sexually transmitted infections and induced abortions results in an overall reduction of EUP rates (Ali, 2016). Human Immunodeficiency Virus (HIV) is known to induce changes leading to tubal damage. In line with this fact, we observed a prevalence of 7.4% of our EUP patients infected with HIV. This observation must be stressed as this percentage represents more than two-fold that of the general population (3.4%) (Simsek and Ay, 2016; Miller, 2006; Fleming and Wasseheit, 1999; Paavonen, 1996). Moreover, in our study, the recorded symptoms were in accordance with the classical picture of EUPs. All patients presented amenorrhoea, 67.0 % complained of pelvic pain but paradoxically only 1.0% of patients reported metrorraghia which can be due to a recall bias (Epee-Bekima and Overon, 2013). Most cases of EUPs were in the ampulla, like previously reported in Cameroon (Foumane et al., 2011; Dohbit et al., 2010). The high prevalence of haemorrhagic shock in ruptured EUP is a common feature in resource-poor settings due to delays in diagnosis and medical care (Randriambololona et al., 2012; Balde et al., 2014; Foumane et al., 2011; Dohbit et al., 2010; Kenfack et al., 2012; Omokanye et al., 2013; Cornelius et al., 2013). This explains why EUP is a big contributor to maternal mortality in Cameroon and thus diagnosis and managing are in dire need of improvement (Kamga et al., 2017).

Like in other hospitals, if laparoscopy is not available, surgical management was done via laparotomy for salpingectomy interventions (Sy et al., 2009). Rupture of EUP and subsequent blood transfusion can be avoided with early diagnosis. Indeed, early diagnosis allows non-surgical management or conservative surgical management (preservation of the fallopian tube). This optimal situation will be difficult to reach in Cameroon because women usually start antenatal care around the fourth month of pregnancy (Tolefac et al., 2017). Importantly, in this study, the success rate (66.7%) of medical management is difficult to appreciate because of the small number of patients treated (Foumane et al., 2011).

In conclusion, the observed EUP rate in Yaounde is high. Single and young women with few or no children are the most affected. Most women diagnosed with EUP have history of sexually transmitted infection and induced abortion. Late diagnosis hinders non-surgical management of EUP as it is most often discovered after rupture and haemorrhagic shock. In view of reducing this burden, women should be sensibilized to start antenatal consultations early and to seek medical care at the onset of symptoms.
